# Motor and Respiratory Tele-Rehabilitation in Patients with Long COVID-19 after Hospital Discharge: An Interventional Study

**DOI:** 10.3390/life14070864

**Published:** 2024-07-10

**Authors:** Serena Cerfoglio, Federica Verme, Paolo Capodaglio, Paolo Rossi, Viktoria Cvetkova, Gabriele Boldini, Manuela Galli, Veronica Cimolin

**Affiliations:** 1 Department of Electronics, Information and Bioengineering, Politecnico di Milano, 20133 Milan, Italy; serena.cerfoglio@polimi.it (S.C.); manuela.galli@polimi.it (M.G.); veronica.cimolin@polimi.it (V.C.); 2 Orthopaedic Rehabilitation Unit and Research Laboratory in Biomechanics, Rehabilitation and Ergonomics, San Giuseppe Hospital, IRCCS Istituto Auxologico Italiano, 28824 Piancavallo, Italy; f.verme@auxologico.it (F.V.); gabrieleboldini@gmail.com (G.B.); 3 Department of Surgical Sciences, Physical Medicine and Rehabilitation, University of Turin, 10126 Turin, Italy; 4 Clinica Hildebrand, Centro di Riabilitazione Brissago, CH-6614 Brissago, Switzerland; p.rossi@clinica-hildebrand.ch (P.R.); v.cvetkova@clinica-hildebrand.ch (V.C.)

**Keywords:** COVID-19, long COVID, rehabilitation, tele-rehabilitation, motor rehabilitation, respiratory rehabilitation

## Abstract

The persistence of symptoms following COVID-19 infection represents a significant challenge in healthcare management. During the outbreak, tele-rehabilitation emerged as a new tool to support healthcare structures in providing rehabilitation services. This study assessed the effectiveness and the feasibility of a 3-week home-based motor and respiratory rehabilitation program for individuals with long COVID-19 after traditional rehabilitation. Twenty-three patients completed the program and underwent functional tests at different time points (i.e., baseline, at discharge from in-hospital rehabilitation and after tele-rehabilitation). Motor function was evaluated using the instrumented Six-Minutes Walking Test (i6MWT), with monitored heart rate and oxygen saturation. Additionally, respiratory function was measured via forced vital capacity (FVC) and maximal voluntary ventilation (MVV) tests. Significant improvements (*p* < 0.05) in motor and respiratory function were observed throughout the intervention, including an 18.3% increase in walked distance from the baseline. The findings suggest that the proposed home-based tele-rehabilitation shows potential in enhancing motor and respiratory function in patients with long COVID. Despite limitations such as the small sample size, lack of control group and the preliminary nature of the outcomes observed, the overall findings seem to support the feasibility of the proposed tele-rehabilitation program in managing long COVID symptoms and promoting functional recovery. Nevertheless, further research is needed to validate these findings and explore tele-rehabilitation’s potential in broader and different patient populations

## 1. Introduction

“Long COVID”, also known as “post-COVID syndrome”, is a term used to describe presence of various symptoms persisting weeks or months after acquiring SARS-CoV-2 infection, and it can affect anyone exposed to the virus, regardless of age or severity of original symptoms [1,2,3]. The World Health Organization (WHO) defines it as the condition occurring in individuals with a history of probable or confirmed SARS-CoV-2 infection, typically commencing three months post-onset of COVID-19 and which symptoms last for at least two months and cannot be attributed to an alternate diagnosis [4,5,6].

The prevalence of long COVID symptoms recorded varies depending on the study subjects and methods, but it has been reported that 10–20% of individuals experience a variety of mid- and long-term effects after they recover from their initial illness [1]. The COVID-19 pandemic has brought significant challenges to the global healthcare system, both in managing patients in the disease’s acute phase, but also in treating COVID-19 patients who have experienced symptoms lasting over time [2,3]. Long COVID can present with several symptoms: the most common include fatigue, breathing difficulties, dyspnoea, muscle weakness and headache [4,7,8] but also mental disorders (i.e., attention deficit, sleep disturbance, depression), olfactory and gustatory dysfunction, and some more severe symptoms affecting respiratory, cardiovascular and gastrointestinal systems have been reported [4,7,9]. There is evidence that these long-term symptoms can also occur in people who have mild or no symptoms during acute infection [10,11], resulting in a lower quality of life and in greater difficulties to perform daily living activities (i.e., walking, climbing stairs or lifting) [8].

Long COVID-19 syndrome has significantly impacted global health, affecting an estimated 65 million people [12,13], and it has been recognized as a public health problem requiring appropriate rehabilitation interventions from the acute to the post-acute phase [5,14,15]. Rehabilitation plays a key role in the functional recovery of patients with long COVID-19 and should be targeting mainly the respiratory and motor symptoms [5]. Patients who have been infected with COVID-19 often complain of fatigue and muscle weakness, making it necessary for them to participate in targeted and interactive programs focused on improving their physical activity levels [3].

Since COVID-19 infections have been linked to respiratory problems [16,17], respiratory rehabilitation is a desirable treatment approach for these patients, as pulmonary rehabilitation has been shown to be effective in improving respiratory function in several respiratory diseases, such as chronic obstructive pulmonary disease (COPD) or asthma [18,19,20,21,22]. However, traditional pulmonary rehabilitation has still been grossly underutilized: it was recently shown in a prospective multi-center cohort that only 1% of patients initiated traditional pulmonary rehabilitation following hospital admission with COPD exacerbation, and the results of previous studies are in line with these findings [23,24], making it necessary to find new therapeutic options to overcome these barriers.

During the COVID-19 outbreak, the access to traditional rehabilitation had to be rethought. Many components of rehabilitation do indeed require patient contact, but the physical distancing for protection of both healthcare workers and patients could result in increased disability and morbidity due to the lack of necessary rehabilitation services [25]. To cope with this new situation, the use of tele-rehabilitation was introduced as a strategy to provide rehabilitation services remotely, with the aim of improving and facilitating access to rehabilitation [25].

Previous research has demonstrated the beneficial effects of tele-rehabilitation and tele-coaching on patients with idiopathic pulmonary fibrosis and chronic obstructive pulmonary disease (COPD) in terms of symptom severity reduction, increased physical activity and exercise capacity and health-related quality of life [3,26,27,28]. Furthermore, according to a recent systematic review, tele-rehabilitation could be an effective tool for the treatment of persistent symptoms in patients who suffered from COVID-19 infection, improving their physical performance and quality of life [29]. Even though the COVID-19 pandemic lockdowns are currently over, health systems are encouraged to adopt telemedicine practices for strengthening healthcare delivery, and tele-rehabilitation may still be regarded as a practical way to care for patients since it is a practical and easily accessible tool that enables long-distance communication and follow-up via information and communication technology (i.e., telephone, internet-based applications and mobile applications) [8,25,30]. Moreover, according to a recent Cochrane review [31], many patients see tele-rehabilitation as affordable and cost saving if the equipment and infrastructure have been provided.

Taking all these aspects into account, the aim of this study was to evaluate the effectiveness of a home-based motor and respiratory tele-rehabilitation program on the recovery of functional exercise capacity in hospitalized patients with long COVID.

## 2. Materials and Methods

### 2.1. Participants

This study addressed a group of individuals suffering from persistent disabling motor and/or respiratory symptoms (e.g., reduced muscular strength, joint and muscle pain, dyspnea and asthenia) after the acute phase of SARS-CoV-2 infection. The eligibility criteria included adult participants of both sexes, ranging in age from 35 to 75 years, who were admitted to the functional recovery units of San Giuseppe Hospital (IRCCS Istituto Auxologico Italiano, Piancavallo, Italy) and Clinica Hildebrand—Centro di Riabilitazione Brissago (Brissago, Switzerland) due to persistent (>3 months) respiratory and musculoskeletal symptoms following SARS-CoV-2 infection. Conversely, individuals presenting uncontrolled cardiovascular diseases, cognitive impairments or psychiatric pathologies preventing the correct use of technology for tele-rehabilitation were excluded. Approval for the study was obtained from Ethics Research Committees of both Institutes (trial registration: NCT05739552), and all procedures were conducted in accordance with their ethical guidelines as well as with the principles outlined in the 1964 Helsinki Declaration and its later amendments. Written informed consent was obtained from all participants prior to their involvement in the study.

All collected personal data and records underwent an anonymization and protection process. All identifiable information was either removed or encrypted to ensure anonymity. Access was restricted to authorized personnel to ensure data privacy and confidentiality.

### 2.2. Therapeutic Intervention

Patients admitted to the functional recovery units followed a four-week multidisciplinary rehabilitation program including physiotherapy, physical activity and conditioning sessions, breathing exercises, medical support and nutritional intervention. Upon discharge, patients were provided with a personalized three-week motor and respiratory tele-rehabilitation program based on individual assessments and hospital rehabilitation course, along with proper, easy-to-use technological equipment for remote rehabilitation. During their hospital stay, patients were instructed on how to use the technologies and become accustomed to them.

In particular, the technology provided to each patient consisted of a respiratory muscle training device (SpiroTiger, MVM Italya, Lainate, Italy) and a motor rehabilitation device (Euleria home, Euleria Health, Rovereto, Italy).

**Figure 1:**
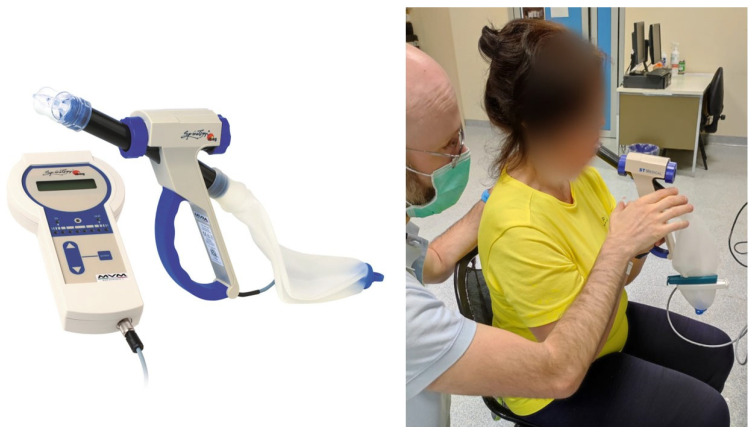
Visual representation of the SpiroTiger device (**left**). A patient training with the device (**right**).

The SpiroTiger is a device specifically designed to train respiratory muscles allowing for the execution of deep and fast breathing acts without incurring the consequences of hyperventilation, such as hypocapnia. The device (Figure 1) consists of a portable grip housing monitoring electronics and a small display. It is fitted with a silicone breathing bag for air recycling, a mouthpiece oriented at 90 degrees and a lateral port enabling fresh air inspiration and expiration. Additionally, the port incorporates a valve regulating the maintenance of the isocapnic state. The tuning of the specific parameters of the device allows for a personalized respiratory training by enabling maximal inspirations and expirations without the risk of hyperventilation, eliciting activation of all respiratory muscle groups, thereby enhancing thoracic flexibility and breathing coordination [32].

Concerning motor training, euleria home is a CE class 1/m medical device designed for home-based rehabilitation. It consists of an IMU (Xsens DOT, Movella Technologies-NL, size: 36.3 × 30.4 × 10.8 mm, weight: 11.2 g) wirelessly connected to a tablet running a dedicated application that guides the user through the execution of a set of motor tasks selected by clinicians from a wide integrated library. The sensor can be easily worn on different body segments with adjustable elastic bands. Once it is in place, the user simply needs to start the application on the tablet and follow the audio-video cues on the screen to perform the exercises. Biofeedback technology and integration with the sensor simplify the execution of the tasks and the completion of the session, while also motivating the patient with an effective gamification mechanism. Through a cloud-based management system, clinicians can remotely monitor patients’ compliance with rehabilitation and adjust personalized exercise therapy programs according to patients’ health status. Additionally, the system facilitates communication with patients through its integrated chat and video call functions.

**Figure 2:**
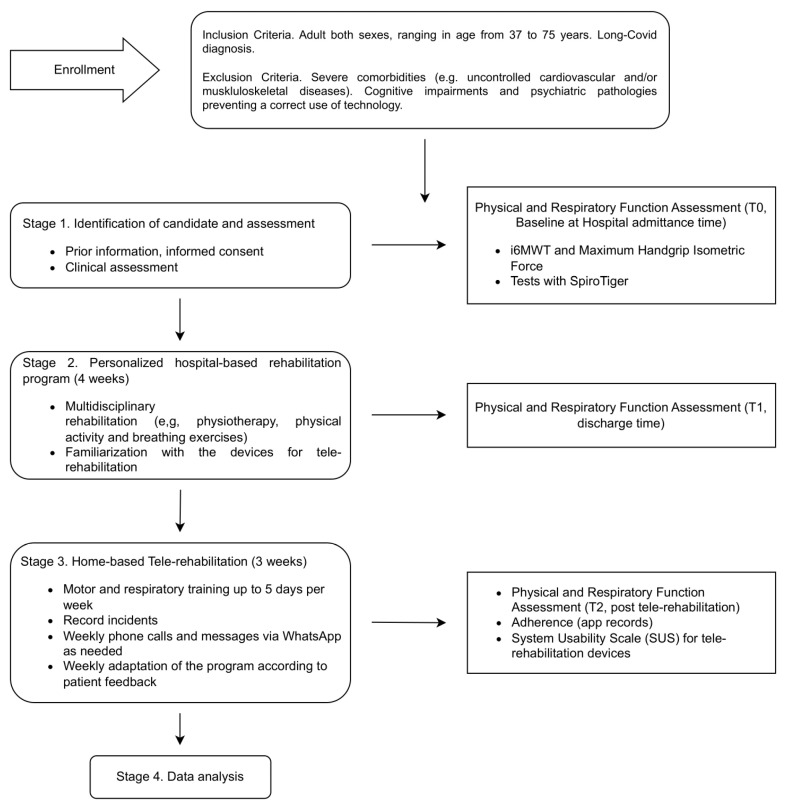
Study workflow.

The exercise protocol for tele-rehabilitation was sorted by clinicians based on individual assessment of the patients prior to hospital discharge. For respiratory rehabilitation, patients engaged in personalized breathing exercises up to five times per week. Conversely, motor rehabilitation mainly included simple free body exercises targeting trunk, upper and lower limbs, to be performed three to five times per week. On a weekly basis, clinicians reviewed the exercise reports to monitor and adjust the program as needed. Since the SpiroTiger does not allow for remote monitoring of the performed session, weekly phone calls were made to evaluate user tracking. In addition, daily monitoring of the program was performed via WhatsApp, and any deviations from adherence was noted, specifically recording any adverse incidents. At the end of the program, participants completed a satisfaction questionnaire (System Usability Scale, SUS) [33] regarding the use of both devices. A flow diagram of the study is shown in Figure 2.

### 2.3. Functional and Clinical Assessment

Post-COVID patients often experience a downward spiral, leading to reduced functional abilities to perform functional tasks due to breathing difficulties, early fatigue and muscle weakness [10,34].

To track and highlight changes in their health status throughout the rehabilitation process, patients underwent functional tests for motor and respiratory function at different time points. Specifically, patients were evaluated at the beginning of their hospital-based rehabilitation program (T0), at discharge from in-hospital rehabilitation (T1), and at the end of their home-based tele-rehabilitation program (T2). All assessments were performed in the hospital setting, using the same set-up and instrumentation to guarantee homogeneity for all time point evaluations.

Regarding motor function, patients performed the instrumented Six-Minutes Walking Test (i6MWT) [35,36,37], a common sub-maximal test used to assess aerobic capacity and endurance in patients with various diagnoses, including those with cardiopulmonary issues [38]. The i6MWT was recorded using a single miniaturized inertial sensor (70 mm × 40 mm × 18 mm) (G-Sensor^®^, BTSBioengineering, Milan, Italy) placed at the L4–L5 vertebrae level with an ergonomic belt. The device, previously validated for gait investigations in both unaffected individuals and those with various pathological conditions [36,39,40,41], integrates a triaxial accelerometer 16 bit/axes with multiple sensitivity (±2, ±4, ±8, ±12 g), a triaxial gyroscope 16 bit/axes with multiple sensitivity (±250, ±500, ±1000, ±2000 °/s) and a triaxial magnetometer 13 bit (±1200 mT).

The i6MWT was conducted indoors along a flat 30 m hospital corridor. Regular 3 m intervals along the corridor were marked with colored tape, while two cones defined the turnaround points. Patients were instructed to walk as fast as possible at their own pace. Rest was permitted as needed, and verbal cues to cues to motivate the patients were provided in accordance with the guidelines from the European Respiratory Society and the American Thoracic Society [42,43]. All assessed patients completed the test without reporting adverse events such as chest pain, severe dyspnea or other signs of severe distress. Heart rate and oxygen saturation (SpO_2_) were measured using a fingertip pulse oximeter both before and after the test.

At the end of the i6MWT, acceleration data (sample frequency: 100 Hz) were wirelessly transmitted to a laptop and processed using a dedicated software (BTS^®^ G-Studio, BTS Bioengineering S.p.A.; v3.2.20) for data collection, elaboration and reporting. The distance covered over 6 min (i.e., walked distance), which typically serves as the primary outcome measure for assessing changes in patients’ performance, was retrieved and considered for further analysis. Additionally, other relevant gait spatio-temporal parameters including gait speed, cadence and stride length, were also recorded. Furthermore, since patients with long COVID are often characterized by compromised muscle strength, also affecting upper limbs and grip abilities [44], upper limb strength and function were evaluated by measuring the maximum isometric force exerted during handgrip for both the dominant (D) and non-dominant (ND) upper limb using a hand-held dynamometer.

With regard to respiratory function, two 1 min maximal voluntary ventilation (MVV) tests and the forced vital capacity (FVC) assessment were conducted using the SpiroTiger device. In particular, the first test utilized a ventilation frequency of 25 acts/min (MVV @25) to specifically load inspiratory muscles, whilst the second test targeted expiratory muscles with ventilation frequency of 34 acts/min (MVV @34) [45]. The recorded FVC, FVV @25 and FVV @34 were taken into account for further analysis.

### 2.4. Statistical Analysis

Descriptive statistics were employed to characterize the sample in terms of age, sex and individual variables such as height and weight. Data from motor and respiratory assessment were checked for normal distribution and homogeneity of variance using the Shapiro–Wilk and Levene’s test, respectively.

To evaluate and quantify the effect of rehabilitation and tele-rehabilitation, a comparison across the three recorded testing sessions (i.e., T0, T1 and T2) was conducted via ANOVA analysis, followed by post hoc analyses including paired t-tests between the observed time points (i.e., T1–T0, T2–T1, T2–T0).

## 3. Results

The two rehabilitation centers evaluated 40 patients from 16 May 2022 to 15 August 2023. Out of the assessed patients, 12 did not meet the inclusion criteria or refused the program. As a result, a total of 28 patients with long COVID started the program, but only 23 completed the intervention and were included in the analysis. Five participants withdrew from the study due to personal reasons unrelated to the rehabilitation program. All participants were diagnosed with SARS-CoV-2 infection between March 2020 and April 2022. The interval between the acute infection and the start of rehabilitation ranged from 4 to 24 months, consistent with the diagnostic criteria for long COVID syndrome. Of the total sample, 11 patients experienced severe infection with interstitial pneumonia and/or respiratory failure requiring hospitalization during the acute phase, while the remaining participants received pharmacological treatment at home.

**Figure 3:**
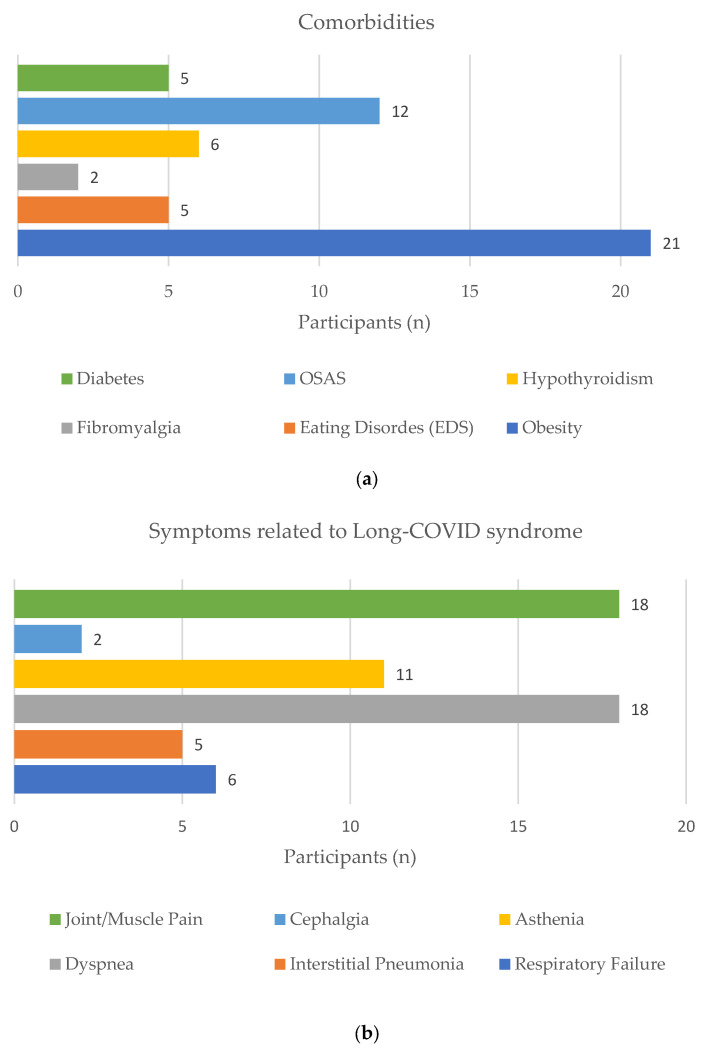
Participants’ comorbidities (**a**) and symptoms related to long COVID syndrome (**b**).

The participants’ characteristics, comorbidities and symptoms related to long COVID syndrome are summarized in Table 1 and Figure 3. It should be noted that the most common comorbidities were obstructive sleep apnea syndrome (OSAS) and obesity. With respect to the latter, at the beginning of the rehabilitation program, only two patients could be categorized as normal weight and overweight, respectively, according to their BMI, while the other 21 patients showed different degrees of obesity.

**Figure 4:**
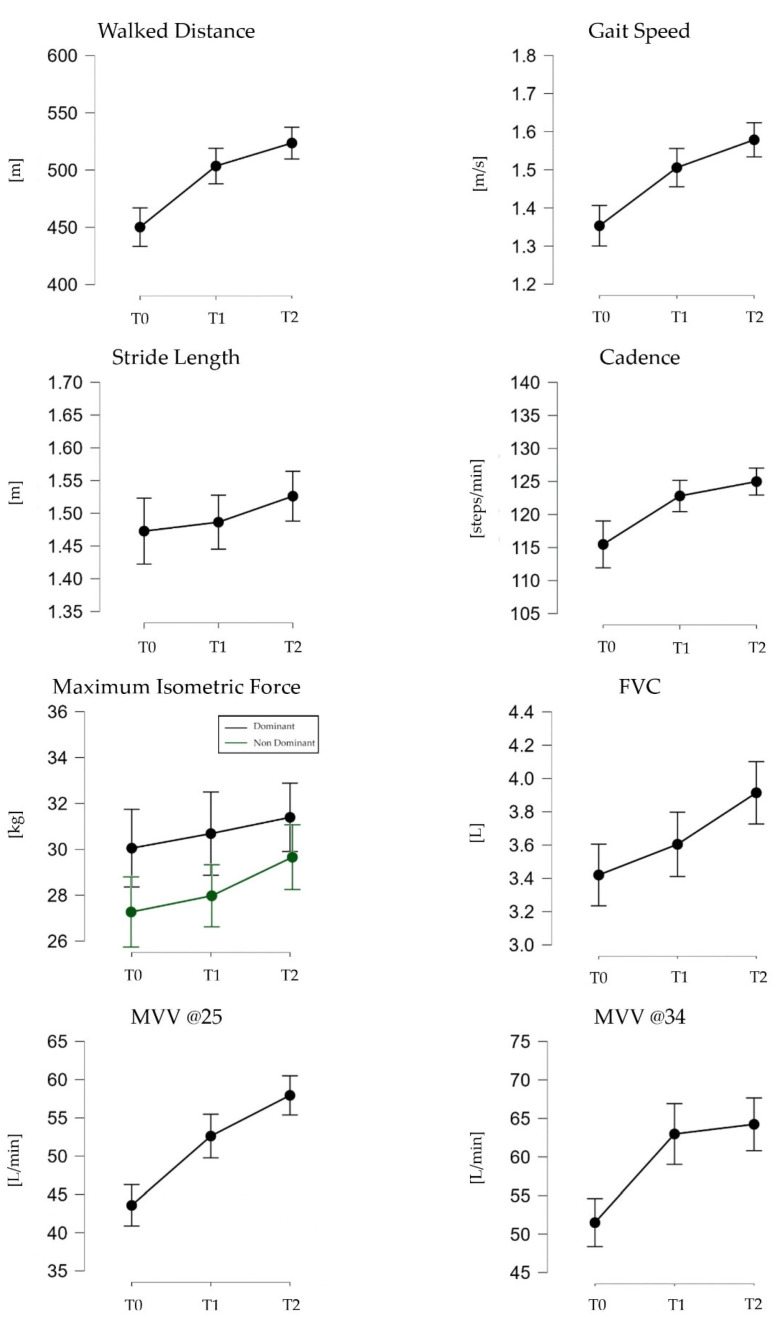
Visual representation of the average values and standard errors of physical and respiratory parameters for each session (T0, T1 and T2).

Shapiro–Wilk test confirmed the normality of the distribution, while the homogeneity of variances was confirmed by Lavene’s test for all parameters, allowing their representation in terms of mean and standard deviation. Inter-session variations for gait spatio-temporal parameters were all found to be significant (*p* < 0.05) between T0 and T1, except for stride length, while cadence appeared to be significant only between T0 and T2. Although the variation between T1 and T2 was not significant, a consistent increasing trend could be observed for all parameters, indicating constant improvement. Conversely, no significant variation was found for the maximum isometric force values, both for the dominant and non-dominant upper limb. Regarding respiratory parameters, significance (*p* < 0.05) was found in all inter-session variations. In particular, the MVV test reported statistically significant (*p* < 0.05) variations both between T0 and T1 (i.e., MVV @25 and MVV @34) and T2 and T1 (i.e., FVV @34), while FVC variation was reported to progressively increase and reach significance (*p* < 0.05) at the end of the whole rehabilitation course (i.e., T2). Motor and respiratory parameters are summarized in Table 2 and in Figure 4.

At the end of the program, patients completed the satisfaction questionnaire. The mean SUS scores were 85.5/100 for the respiratory training device and 84.1/100 for the motor training device, respectively.

Table 3 reports the intra-session changes in heart rate and oxygen saturation before and after the execution of the i6MWT at each time point. A significant (*p* < 0.05) variation was found in the pre-post test heart rate in all sessions, whilst a significant variation for SpO_2_ was observed only at the baseline session. No significant (*p* > 0.05) inter-session variations were found. However, it is noteworthy that the intra-session variation decreases for SpO_2_, indicating changes in patients’ oxygen desaturation after the test.

## 4. Discussion

COVID-19 patients often experience a heterogenous sequelae of long-term health issues that can persist for months after the infection, hindering their return to normal life and negatively impacting both their mental and physical well-being. Managing long COVID symptoms and effects is crucial across all stages of the disease, from the acute to post-acute period. However, access to rehabilitation facilities was often limited during the pandemic outbreak. In this context, tele-rehabilitation emerged as a novel solution to support healthcare facilities in coping with the demand for rehabilitation, not only for chronic patients, but also for COVID-19 survivors. Therefore, this study aimed to assess the effectiveness of a home-based tele-rehabilitation program tailored for patients with long COVID, with a specific focus on motor and respiratory training.

The results demonstrate an overall improvement in both motor and respiratory function, observed between the baseline assessment at T0 and the completion of hospital-based rehabilitation at T1, as well as between T0 and after tele-rehabilitation at T2. Regarding motor evaluation, the spatio-temporal parameters of gait derived from the i6MWT exhibit a significant (*p* < 0.05) upward inter-session trend, with the exception of stride length. In the traditional 6MWT assessment, walked distance serves as the benchmark for comparing changes in patients’ exercise tolerance, with increased distance indicating improved basic mobility [46,47]. The literature suggests that a minimum inter-session difference of 30 m should be observed to indicate a clinically relevant change in patients with respiratory diseases [42]. In this study, the walked distance increased by approximately 73 m between the baseline and final assessment, with a more pronounced variation observed between baseline and hospital-based rehabilitation (>50 m) compared to tele-rehabilitation (<25 m). Alongside this variation, there is an increase in the mean pre-post test heart rate, consistent with the variation in walked distance [48]. Moreover, in T1 sessions, it is noticeable that SpO_2_ has a reduced decrease after the i6MWT with respect to T0 session. SpO_2_ represents the percentage of hemoglobin in the blood saturated with oxygen. During exercise, since the body’s demand for oxygen increases, SpO_2_ typically decreases because more oxygen is used by muscles and tissues [49,50]. A decrease in the difference between pre- and post-exercise SpO_2_ levels thus suggest a reduced tendency for oxygen desaturation after physical exertion, which is indicative of improved efficiency in oxygen consumption and cardio-pulmonary fitness resulting from rehabilitation. Conversely, a slight increase in the pre-post SpO_2_ variation can be observed at T2. However, it should be acknowledged that for the final assessment, most patients returned to a clinical center which is situated approximately 1300 m above sea level, potentially influencing the saturation value due to altitude changes [51]. Beyond walked distance, it should be noticed that the i6MWT was performed with a body-worn device allowed for the acquisition of other relevant gait parameters, contributing to a more comprehensive understanding of patients’ functional exercise capacity and gait [52]. Previous studies have highlighted the influence of the interplay between cadence and stride length on gait speed, impacting critical spatio-temporal aspects of walking and motor control [53,54,55]. Gait speed can be adjusted by modifying either cadence, stride length or both simultaneously [56]. In this study, although the variation in stride length did not reach statistical significance (*p* > 0.05), the observable upward trend, in line with the rise in cadence, suggests enhanced motor control aimed at augmenting gait speed to increase walked distance during the test.

Regarding the maximum isometric force, an increase between sessions is observable for both dominant and non-dominant upper limbs, although no significant difference (*p* > 0.05) was found. Typically, improvements in muscle strength become noticeable within a few weeks of a structured training program. However, significant changes usually become more evident after several weeks to a few months of consistent training. In this study, the rehabilitation regimen did not specifically target strength training, and the time between sessions may not have been sufficient to observe visible changes in strength [57]. However, the observed differences may reflect general improvements in overall physical condition rather than specific gains in muscle strength. Future studies with longer intervention periods or targeted strength training protocols may provide further insights into the effects of rehabilitation on muscle strength in post-COVID patients.

Significant improvements were reported for all the considered respiratory parameters. FVC is a proxy of lung functional capacity because it quantifies the ability to inhale and exhale. In healthy adults, it typically ranges between 3.0 and 5.0 L, with diminished values often associated with diseases impacting lung function, such as COVID-19. In this study, patients initially exhibited borderline FVC values, which significantly and progressively improved after the hospital- and home-based rehabilitation. Simultaneously, a statistically significant (*p* < 0.05) and coordinated improvement in inspiratory and expiratory muscle strength was observed, as indicated by the results of the maximal 1 min ventilation tests. The respiratory system relies on the interplay of several muscles to facilitate breathing. Therefore, a simultaneous increase in both inspiratory and expiratory muscle strength can lead to improved respiratory function, increased lung capacity and overall breathing performance, as well as reduced fatigue [58,59].

With respect to tele-rehabilitation, the aggregated findings of this study appear promising in supporting both the feasibility and the safety of the approach, as well as its role in enhancing functional exercise capacity and managing long-lasting symptoms in post-COVID individuals. Out of the total population, only five patients initially enrolled did not complete the program, resulting in a dropout rate of approximately 18%, consistent with the existing literature for post-COVID individuals, which ranges from 10 to 32% [14]. Among these, three dropped due to a lack of cooperation, while the others withdrew from the program due to personal reasons. Throughout tele-rehabilitation, participants received regular monitoring from healthcare professionals via video calls and messages as needed. This continuous monitoring likely aided patients in maintaining consistency with the training regimen, since dropout in tele-rehabilitation has been reported to stem from patients’ misperceptions of their actual needs and loss of human contact with clinicians [14]. Furthermore, rehabilitation plans were tailored to address the individual needs of each patient and scheduled to accommodate their daily lives and routines, as programs demanding high commitment in terms of both physical effort and time are more prone to dropout. Regarding safety aspects, it is important to note that no adverse events occurred during the home-based program. While the heterogeneity of long COVID outcomes may affect safe participation in tele-rehabilitation [60], the absence of adverse events supports the proposed program as a safe solution for managing patients with long COVID.

Regarding technological aspects, patient feedback on the use of the devices for tele-rehabilitation supports the sustainability of the proposed approach. Patients generally expressed satisfaction and motivation with using the devices at home and being followed up weekly (albeit remotely) by healthcare professionals. This positive feedback was also confirmed by the completion of satisfaction questionnaires, whose SUS scores would suggest exceptional usability for both the devices [61]. Although the introduction of specific technological platforms and devices may pose some challenges and slow down the implementation of tele-rehabilitation [62], particularly for patients unfamiliar with technology, no issues were reported by the participants. The devices were selected for their ease of use and accessibility, and patients had the opportunity to familiarize themselves with the devices during their hospital stays. During tele-rehabilitation, only sporadic technical issues such as disconnections and device failures were reported, although they were promptly addressed by technical support.

While the study highlights positive aspects of the rehabilitation program, several limitations should be taken into account. Firstly, the study population was not consistent due to changes in the pandemic situation in Italy during the study period. The reduced number of participants thus limits the robustness of the statistical findings, which may not be representative of a broader population. Furthermore, most participants had comorbid obesity and OSAS, potentially impacting the generalizability of the findings. Chronic inflammation of adipose tissue alongside with reduced lung capacity and impaired ventilation are reported to increase the vulnerability of obese individuals to severe illness from COVID-19, so it is not unusual to have a high number of patients with comorbid obesity dealing with long COVID symptoms [63,64,65]. In addition, individuals with obesity associated with OSAS may face increased risks if they contract COVID-19, as the intermittent hypoxia characteristics of OSAS may predispose them to an enhanced inflammatory response and more severe respiratory complications [66,67]. However, it is worth mentioning that the clinical center that treated the majority of patients is specialized in the treatment of patients with metabolic disorders, so recruitment was carried out according to the availability of eligible patients regardless of their BMI. However, the recovery mechanisms and timings from COVID-19 may differ for healthy weight individuals [68,69]. In addition, it should be noticed that the number of male and female participants was not equal. This was due to the availability of patients during the study period but also to the fact that female individuals seem more prone to suffer long COVID syndrome [70,71]. Furthermore, since recovery mechanisms may also differ between genders [72], understanding and assessing these differences may be important for tailoring effective rehabilitation strategies for these patients.

Another limitation is the absence of a control group, as reported in previous studies [5,73,74]. Without a control group, caution is needed in interpreting the data, as distinguishing whether improvements are due to the rehabilitation course or the natural evolution of the disease over time may be challenging [73,74]. Moreover, since patients underwent hospital-based before tele-rehabilitation, quantifying their respective impacts on patients’ recovery is difficult. Also, the absence of a gold standard method for assessing respiratory capacity, such as the spirometry test, limits generalizability of results. However, it is worth noticing that the respiratory improvements observed in this study align with findings from a previous prospective study involving a large sample of individuals with long COVID syndrome [75], which reported no change, for example, in FVC between 3 and 6 months after acute COVID infection. In contrast, the current study observed consistent and progressive improvements throughout the rehabilitation course, suggesting they could be related not only to the time elapsed since the acute infection, but also the rehabilitation intervention.

This study employed a hybrid approach combining traditional face-to-face rehabilitation and tele-rehabilitation, ultimately leading to positively synergic effects on the outcomes. Throughout the pandemic period, the demand for rehabilitation services has increased significantly. Tele-rehabilitation is not meant to fully replace traditional rehabilitation, rather, its implementation offers a viable new approach balancing patient quality of life and healthcare system sustainability [76,77,78]. Besides the observed benefits in motor and respiratory function among program participants, tele-rehabilitation can also yield socio-economic advantages for both patients and healthcare facilities. Utilizing digital technology to deliver rehabilitation has the potential to reduce hospital stay, decrease the demand for human resources and lower hospitalization costs [79,80,81]. However, cost-effectiveness studies are still needed.

## 5. Conclusions

The aim of this study was to evaluate the effectiveness of a therapeutic intervention delivered via tele-rehabilitation in facilitating motor and respiratory recovery among individuals with long COVID. Despite the discussed limitations, the overall outcomes seem to suggest the potential clinical benefits of this approach in promoting recovery in patients with long COVID, along with positive adherence and safety aspects. Given the current COVID-19 diffusion and its evolving landscape, enlarging the patient population or incorporating a control group poses significant challenges. Nevertheless, there might be the potential to extend the tele-rehabilitation protocol to individuals with other conditions affecting respiratory and/or motor functions. Given the outcomes and their implications from both patient and healthcare center perspectives, the tele-rehabilitation approach proposed in this study appears to offer a potentially viable, safe and effective strategy in addressing rehabilitation needs. This could open up new perspectives on tele-health applications in supporting healthcare centers in the provision of rehabilitation services.

## Figures and Tables

**Table 1:** Demographic characteristics of the post-COVID patients who completed the program. Values are expressed as mean (SD).

	Participants (n = 23)
M/F	6/17
Age (years)	55.91 ± 7.57
Height (cm)	164.58 ± 6.20
Weight (kg)	98.11 ± 17.82
BMI (kg/m^2^)	36.38 ± 7.06

**Table 2:** Mean and standard deviation of the parameters related to motor and respiratory assessment. * = *p* < 0.05, if T0 vs. T1 and/or T2; ^+^ = *p* < 0.0, if T1 vs. T2.

	Parameter	Sessions		
Motor Assessment		T0	T1	T2
	Walked Distance (m)	450.16 (69.24)	503.52 (63.85) *	523.59 (57.59) *
	Gait speed (m/s)	1.35 (0.22)	1.51 (0.20) *	1.58 (0.18) *
	Stride Length (m)	1.47 (0.21)	1.49 (0.16)	1.53 (0.16)
	Cadence (steps/min)	115.48 (14.59)	122.81 (9.44)	124.99 (8.43) *
	Maximum Isometric Force (kg) (D)	30.05 (7.56)	30.69 (8.53)	31.40 (6.81)
	Maximum Isometric Force (kg) (ND)	27.39 (8.05)	28.22 (7.48)	30.20 (7.62)
Respiratory Assessment	FVC (L)	3.42 (0.81)	3.60 (0.91)	3.91 (0.88) *^,+^
	MVV @25 (L/min)	43.57 (12.09)	52.61 (12.38) *	57.93 (12.02) *^,+^
	MVV @34 (L/min)	51.49 (12.89)	62.97 (18.54) *	64.23 (16.09) *

**Table 3:** Mean and standard deviation of heart rate and SpO_2_ before and after the i6MWT. * = *p* < 0.05, pre-test vs. post-test in each session (i.e., T0, T1 and T2).

	Heart Rate (bpm)		SpO_2_(%)	
Session	Pre-test	Post-test	Pre-test	Post-test
T0	76.39 (11.55)	101.41 (17.14) *	95.33 (2.45)	91.06 (6.19) *
T1	81.40 (14.52)	108.55 (20.46) *	94.50 (2.36)	93.40 (3.85)
T2	85.05 (17.41)	112.10 (20.04) *	94.05 (5.39)	92.40 (4.13)

## Data Availability

Data are available on request in Zenodo repository (https://doi.org/10.5281/zenodo.11353640) due to restrictions, e.g., privacy or ethical.
